# Neuropeptides Involved in Facial Nerve Regeneration

**DOI:** 10.3390/biomedicines9111575

**Published:** 2021-10-29

**Authors:** Inhyeok Kim, Yonjae Kim, Daewoong Kang, Junyang Jung, Sungsoo Kim, Hwasung Rim, Sanghoon Kim, Seung-Geun Yeo

**Affiliations:** 1 College of Medicine, Kyung Hee University, Seoul 02453, Korea; kiminin2000@naver.com; 2 Department of Otolaryngology—Head & Neck Surgery, School of Medicine, Kyung Hee University, Seoul 02447, Korea; yonjaekim@hotmail.fr (Y.K.); kkang814@naver.com (D.K.); marslover@naver.com (H.R.); hoon0700@naver.com (S.K.); 3 Department of Anatomy and Neurobiology, School of Medicine, Kyung Hee University, Seoul 02447, Korea; jjung@khu.ac.kr; 4 Department of Biochemistry and Molecular Biology, School of Medicine, Kyung Hee University, Seoul 02447, Korea; sgskim@khu.ac.kr

**Keywords:** neuropeptides, facial nerve, regeneration

## Abstract

Neuropeptides and neurotransmitters act as intermediaries to transmit impulses from one neuron to another via a synapse. These neuropeptides are also related to nerve degeneration and regeneration during nerve damage. Although there are various neuropeptides, three are associated with neural regeneration in facial nerve damage: calcitonin gene-related peptide (CGRP), galanin, and pituitary adenylyl cyclase-activating peptide (PACAP). Alpha CGRP in facial motoneurons is a signaling factor involved in neuroglial and neuromuscular interactions during regeneration. Thus, it may be a marker for facial nerve regeneration. Galanin is a marker of injured axons rather than nerve regeneration. PACAP has various effects on nerve regeneration by regulating the surrounding cells and providing neurotrophic factors. Thus, it may also be used as a marker for facial nerve regeneration. However, the precise roles of these substances in nerve generation are not yet fully understood. Animal studies have demonstrated that they may act as neuromodulators to promote neurotrophic factors involved in nerve regeneration as they appear early, before changes in the injured cells and their environment. Therefore, they may be markers of nerve regeneration.

## 1. Introduction

Facial nerve paralysis can be caused by a variety of conditions, including idiopathies, infection (such as Ramsay Hunt syndrome, otitis media with effusion, acute suppurating otitis media, chronic otitis media, malignant otitis externa, tuberculosis, Lyme disease, and AIDS), trauma (including temporal bone fracture, birth damage, facial lacerations, penetration damage, iatrogenic damage, and radiation injury), tumor (such as cholesteatoma, glomeruloma, malignant tumor, facial neurinomas, meningioma, histiocytosis, and rhabdomyosarcoma), congenital (including crush injuries, Möbius syndrome, and labialis inferior palsy), recurrent (including repetitive facial palsy and Melkersson-Rosenthal syndrome), and metabolic and systemic (such as sarcoidosis, Guillain–Barre syndrome, and autoimmune diseases) [1,2] (Table 1).

While facial paralysis is not a life-threatening condition, if recovery is incomplete, patients will experience psychological and social deterioration, which can impact social activities; therefore, complete treatment is important [3,4]. However, although various studies have been conducted on the cure of facial paralysis, no specific biomarkers or complete remedies have been established and the exploration of new materials for neuroregeneration has been slow.

Like any motoneurons of the peripheral nervous system, injured facial nerves in adults can regenerate. Efficient remyelination and axonal sprouting of the appropriate target muscles are needed for morphological recovery [5]. However, the molecular processes involved in nerve regeneration are complex and not yet completely understood. A close interaction between the injured motoneuron and its surrounding non-neuronal cells (periglia, T lymphocytes, and endoneurial fibroblasts) promotes cell differentiation, cytoskeletal reorganization, axonal guidance, and angiogenesis [6]. Among glia, Schwann cells form the myelin sheath surrounding the axon, produce neurotrophic and growth factors, and generate extracellular matrices as scaffolding for axonal sprouting. Astrocytes create glial filaments that provide trophic support for axonal outgrowth. Resting microglial cells migrate to the site of injury and produce neurotoxic factors to remove debris from dying cells. Metabolic and morphological changes also occur in the facial motor nucleus [7]. The molecules involved in neurite growth are also upregulated to ensure synaptogenesis. However, other molecules involved in synapsis neurotransmission are downregulated. Among the numerous synthesized molecules, small proteins modulating synaptic transmission, which are neuropeptides, may contribute to axonal sprouting.

Neuropeptides are chemicals that act as intermediaries to transmit impulses from one neuron to another via a synapse. Most neuropeptides are neurotransmitters or neuromodulators involved in the transmission of information in the nervous system; however, like neurohormones, some neuropeptides are directly released from nerve cells into the blood and act on non-neuronal cells to regulate physiological functions. Peptide hormones secreted by endocrine organs are often also produced in the brain. Recently, neuropeptides have been identified that play a wide range of physiological roles in addition to neurotransmission. Representative neuropeptides include substance P, opioid peptides (enkephalin, dynorphin, etc.), neuropeptide Y, neurotensin, oxytocin, vasoconstrictor hormone, somatostatin, cholecystokinin, vasoactive intestinal peptide (VIP), galanin, gastrin-releasing peptide (GRP), calcitonin gene-related peptide (CGRP), neurokinin, neuromedin, and delta-sleep-inducing peptide (DSIP), as well as hypothalamic hormones, thyroid-stimulating hormone-releasing hormone (TRH), luteinizing hormone-releasing hormone (LHRH), adrenocorticotropic hormone-releasing hormone (CRH), and growth hormone-releasing hormone (GHRH) [8,9,10,11]. These neuropeptides may be involved in neurodegeneration and nerve regeneration during nerve damage.

**Figure 1:**
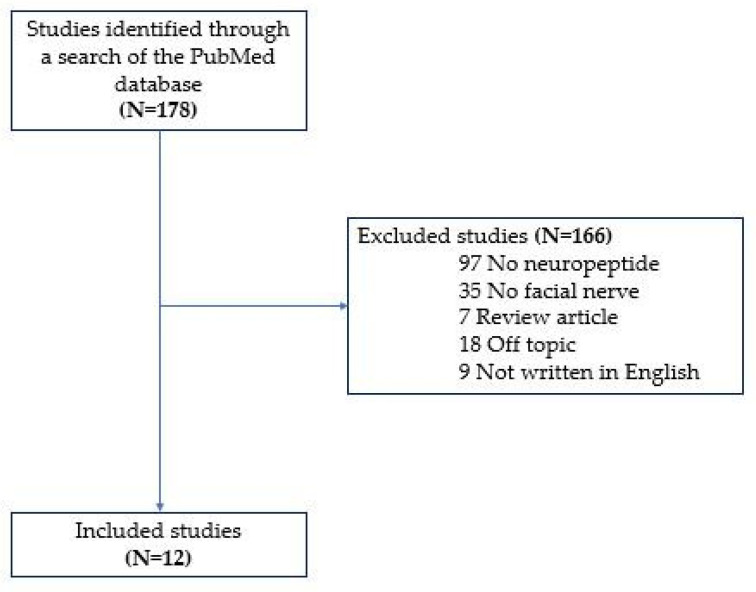
Review flow diagram.

This study aimed to understand neuropeptide expression in facial motor neurons after nerve injury and their specific roles in cell responses to nerve regeneration. We reviewed studies investigating neuropeptides involved in facial motor neurons after axotomy. We identified 178 studies published from 1986 to 2021 indexed in the PubMed electronic database based on the search terms “facial nerve”, “facial motor neurons”, “injured”, “axotomy”, “nerve regeneration”, “molecular expression”, “neuropeptide”, and “neuromodulator”. Studies were conducted on animal models following facial nerve transection or crushing. Neuropeptides were specifically detected in the facial nuclei by immunohistochemistry or in situ hybridization histochemistry, radioimmunoassay, or northern blot analysis. Studies written in languages other than English, review articles, and studies on molecules other than neuropeptides secreted by facial nerve motoneurons were excluded (Figure 1).

## 2. Anatomy and Physiology of the Nervous System and Facial Nerves

### 2.1. Differences between Central and Peripheral Nervous Systems

Neural tissues comprise neurons and neuroglia. Neurons are cells that perform the inherent function of neural tissue that transmits stimuli and are connected through synapses and neurotransmitters to form a complex network. Neuroglia (glias) closely surround nerve cells and perform various functions, including structural support of nerve cells or involvement in the recovery of damaged neurons. These include central and peripheral neuroglia. Central neuroglia are composed of astrocytes, oligodendrocytes, microglia, and ependymal cells. Astrocytes are the largest cells in the group and stretch to enclose blood vessels and nerve cells. The enclosing blood vessels among the protruding ends of astrocytes are involved in maintaining blood-brain barriers and enclosing nerve cells prevents neurotransmitters from spreading beyond the synapse or eliminating excessively secreted neurotransmitters. Oligodendrocytes are responsible for forming myelin sheaths, while microglia are responsible for phagocytosis when there is damage or disease to the central nervous system. Ependymal cells cover the inner surface of the ventricular system to protect and support nerve cells. Similar to oligodendrocytes in the central nervous system (CNS), Schwann cells surround nerve axons of the peripheral nervous system, forming a multi-layered myelin sheath. In addition, satellite glial cells (SGCs) are found exclusively in peripheral ganglia, including sensory, parasympathetic and sympathetic ganglia. SGCs encircle nerve cells in the peripheral nervous system, serving to electrically insulate and mediate material exchange. Dorsal root ganglion (DRG) neurons, which are located in the sensory pathway leading from the skin to the brain, extend two axons, with one central branch extending through the central nervous system and the other peripheral branch extending to sensory organs.

In general, the nerve cells that make up the peripheral nervous system regenerate normally after a certain period if they suffer physical axonal damage. However, damaged nerve cells in the central nervous system will not recover, resulting in permanent failure of function. One of the key factors that limit regeneration of the central nervous system is the formation of ‘scar’ following tissue damage. These scars occur owing to damage to non-neuronal cells before regenerative mechanisms can be sufficiently activated, and thus act as a physical barrier to the regenerative process of reconnecting to the original target. Therefore, full regeneration of the central nerve requires timely inhibition of scar formation with simultaneous full activation of nerve cell regeneration mechanism [12]. Another important factor limiting regeneration of the central nervous system is the fact that proteins such as Nogo, myelin-associated glycoprotein (MAG), and oligodendrocyte myelin glycoprotein (OMgp) produced by astrocytes or meningeal fibroblasts interfere with nerve regeneration [13,14,15]. Genetically, completely removing these major obstructions from rats does not increase the regeneration of the central nerve [16]. In other words, while the presence of regenerative inhibitors is an obstacle to the neuro-renewal process, removing them is not sufficient to promote neural regeneration [12]. In addition to the neuro-renewal inhibitors that appear from the debris of damaged myelin following injury of the central nervous system, chondroitin sulfate proteoglycans (CSPGs) produced by the formation of glial scars are another significant obstacle to neural regeneration [17]. Thus, important remaining research goals in the treatment of facial paralysis include: (1) revealing the biological process that occurs after nerve damage, (2) understanding the neural regeneration process, and (3) developing a treatment method.

### 2.2. Facial Nerve Function and Mechanisms of Nerve Recovery after Injury

Facial nerves are mixed nerves that simultaneously provide motor and sensory functions and are responsible for the movement of the muscles of the face and neck. They also contain parasympathetic nerve components for lacrimal and salivary gland secretions. The facial nerves also include special sensory nerves that sense taste of the front two-thirds of the tongue, as well as general sensory nerves that are responsible for the perception of the auricle, posterior wall of the external auditory canal, ear lobe, and deep perception of facial soft tissue. Thus, the facial nerves comprise two afferent nerves and two efferent nerves, which combined have four functions [1,2,18,19].

If a neuronal cell body is destroyed, it can no longer survive. However, if a portion of the axon is amputated, the neuron can regenerate the axon; moreover, under appropriate conditions, the cell may also restore its synaptic function. Peripheral nerve fibers, in particular, can be regenerated if there is no damage to the cell body. The change in nerve fibers after nerve damage depends on the degree of damage, with local demyelination and remyelination occurring for minor damage such as stage 1 damage and neuropraxia and axonal degeneration, and regeneration for severe damage. In general, facial nerve injury causes axonal injuries that lead to the death of neurons through immediate necrosis or prolonged apoptosis. In cases where the axon is severed, nerve degeneration progresses in opposite directions from the cut: proximal (connected to the cell body) and distal to the opposite directions. The Wallerian degeneration process usually begins 24–36 h after the initial injury, at which point axons start to disintegrate and Schwann cells at the distal end release growth factors [20,21]. Thereafter, calcium influx occurs continuously throughout the cytoplasm and mitochondria via action of the distal pump [22]. Influx of calcium triggers endogenous proteolysis and degeneration of the cytoskeleton [23]. As the process continues, axons start to collapse and Schwann cells lose their myelin sheath. Many cells, including macrophages, are also recruited and serve to remove degenerated axons and myelin debris. A number of factors have been demonstrated to participate in this process, including pro-apoptosis factors, neurotrophic factors, and growth-associated protein (GAP)-43 [24]. Substances transported along the axon accumulate, causing swelling of both cut faces. In the nerve regeneration process, areas of acute severe damage may undergo regeneration after degeneration of the axon. In chronic compressive injury, axon degeneration and regeneration are induced simultaneously. Endoneurial fibroblasts and Schwann cells proliferate and move from the amputated nerve fibers to form a skeleton connecting the damaged site. The Bugner band—a column of Schwann cells—fills the lower endoneural sheath, forming a pathway for axon growth [25,26,27]. Metabolic changes in the cell body also increase synthesis of mRNA, enzymes, and protein that support axon regeneration. Genes encoding proteins such as GAP-43 and cytoskeletal proteins are upregulated [28]. If axons do not regenerate, the Bugner band contracts, Schwann cells decrease in number, and the damaged area is uniformly filled by connective tissue. The axoplasm and neurofilaments swell within hours of damage, with numerous axonal sprouts growing toward the distal area from the upper end of the damaged area 4 days later [28]. The condition of the damaged area determines the direction of regeneration of new axons. Neural recovery occurs actively in the early stages of damage and the thinner the diameter and myelin sheath, the better the recovery. A number of studies investigating facial nerve regeneration have reported that various neurotrophic factors secreted from damaged nerves contribute to the creation of an environment supportive of nerve survival and axon regeneration. In addition, recovery is uneven, with pain sensation showing the best recovery, followed by tactile sensation, proprioceptive sensation, and motor nerve function. Moreover, the younger the age and the closer the nerve end, the better the prognosis. Prognosis is poor in cases where nerve damage is accompanied by soft tissue damage or where motor and sensory nerves are simultaneously damaged [1,29,30,31,32].

## 3. Neuropeptides

### 3.1. General Concept

Neuropeptides are neurotransmitters that are composed of amino acids linked by peptide bonds. They are relatively large and consist of 3–36 amino acids. Neuropeptides are released into the synaptic cleft, along with other neurotransmitters. The body reacts to foreign substances in the neuroendocrine immunologic network (NEI), which comprises three organ systems; namely, the nervous, endocrine, and immune systems. The NEI consists of a well-integrated multidirectional traffic path that connects the three organ systems and is well coupled by transmitters such as neurotransmitters of the nervous system, hormones of the endocrine system, and cytokines of the immune system.

More than 50 types of neuropeptides have been isolated and their structures elucidated (Table 2) [33,34,35,36,37,38,39,40,41,42,43,44]. These are amino acid complexes range in size from two to several dozen, some of which are present in the brain and perform neurochemical, neurophysiological, neuropharmacological, and behavioral functions. With the recognition of the importance of neuropeptides as chemical messengers for neurotransmission or neuromodulation, interest in the role of neuropeptides in various diseases has increased.

### 3.2. Roles of Neuropeptides in Facial Nerve Regeneration

Among various known neuropeptides, three are reportedly related to post-facial nerve regeneration: calcitonin gene-related peptide (CGRP), galanin, and pituitary adenylyl cyclase-activating peptide (PACAP).

#### 3.2.1. Calcitonin Gene-Related Peptide (CGRP)

CGRP is a neuropeptide of 37 amino acids generated by alternative splicing of the calcitonin gene primary transcript. It is widely distributed in the peripheral and central nervous systems. Two subtypes have been identified—α-CGRP and β-CGRP—which differ only by a single amino acid. While they are both expressed in facial motoneurons during nerve regeneration, their reactions to axotomy differ. In spinal motor neurons, α-CGRP mRNA levels increase, while those of β-CGRP mRNA do not change. In the facial motoneurons of rats, β-CGRP mRNA levels decreased after axotomy, whereas those of α-CGRP increased. In rats with nerve transection injury, this difference lasted more than 8 weeks, whereas those with a crush injury showed a temporary change. Subsequent measurements showed normal expression levels during nerve regeneration [45,46].

Experimental studies reported massive and early increases in α-CGRP levels in regenerating motoneurons, whereas β-CGRP levels decreased after a transient early increase. In a mouse model, CGRP immunolabeling intensity was increased in the genu and root of the facial nerve after facial nerve injury. This result indicates that CGRP can be used as a marker for axon regeneration [47]. Moreover, only a slight increase in CGRP mRNA level in the neurons of the unoperated nuclei was noted after axotomy [48]. The increase of factors required for neuronal regeneration and decrease in those related to neuronal transmission after axotomy suggest their different roles. α-CGRP may have a supportive role in neuronal regeneration, whereas β-CGRP may be involved in neural transmission.

A biphasic, five-fold elevation of α-CGRP levels in motoneuron cell bodies following axotomy has been reported [49]. The first increase was transient and was observed very early on Day 3. The level then temporarily decreased on day 9 before showing a second peak on day 21, when nerve regeneration occurred. Neuropeptides are likely newly synthesized in response to the acute loss of trophic factors caused by the disconnection of the neuron from its muscle because denervated muscle fibers inhibit CGRP expression [49]. Peptide levels in axotomized facial nuclei may reflect a disturbed balance between regulatory influences from supranuclear input (CGRP-inducing) and innervated muscle (CGRP-inhibiting). This first peak increase occurs very early in comparison to other structural neuronal proteins, such as tubulin and actin, and before any glial changes. This corresponds to the time to early astrocyte reaction observed in the regenerating facial nucleus. Thus, intracellular CGRP proteins act as neurotrophic factors for glial cell initiation and regulation, particularly for astrocytic reactions during motor nerve regeneration. The second peak occurred on day 21, at the time of reinnervation of the target tissue. While this peak does not occur if the nerve is resected again, after sciatic nerve resection, in the fifth lumbar dorsal root ganglion (L5 DRG), both CGRP mRNA and peptide levels were decreased. Thus, CGRP has different functions depending on the target tissue or neuron type [50]. Depending on the type of nerve injury, the CGRP mRNA expression extent shows different patterns. Similarly, when the nerve is crushed instead of transected with an intact neuromuscular connection, CGRP upregulation is not biphasic but continuous. Therefore, CGRP is a marker of neuromuscular interaction during reinnervation [51]. Finally, the increase persists for several weeks, followed by a gradual decline consistent with the morphological and functional recovery of motor innervation [52]. Only half of axotomized facial motoneurons show increased CGRP mRNA synthesis. The other half of the nuclear cells express galanin [48].

#### 3.2.2. Galanin

Galanin is a 29–30-amino acid neuropeptide that is widely distributed in the peripheral and central nervous systems. However, it is not detected in normal facial nuclei. Several studies reported increased galanin levels in response to axotomy in peripheral sympathetic, peripheral sensory, and peripheral motor neurons, corresponding to approximately 5% to 40–50% increases in neuron number. In the animal model, the average axon length and number of branch points increased in the presence of galanin, indicating its neurotrophic features [52]. Galanin interacts with two G protein-coupled receptors, GalR1 and Gal R2, located in the cell membrane. Whereas GALR1 is present in large-diameter neurons, small- to medium-sized neurons predominantly express GALR2. In addition, GalR1 and GalR2 have different secondary messenger signaling mechanisms. Unlike the sciatic nerve, selective early upregulation of GalR2 has been reported after facial nerve injury [53]. GalR2 levels increased fourfold 1 week after facial nerve crush, indicating its importance as an ‘autoreceptor’ [52]. Galanin promotes axonal growth in the early stages but not in the later events such as muscle innervation. After nerve crush injury, galanin mRNA levels increased for up to 4 weeks and then decreased, with a longer increase period observed for resection injury. This parallel increase of galanin mRNA level to axon regeneration suggests its association with regeneration [45]. In addition, the time course seemed to coincide with central sprouting and neuronal cell death, suggesting a trophic effect on nerve generation [45,46].

However, a recent report indicated that galanin levels returned to normal 1 month after surgery. In that study, galanin levels increased after nerve injury, similar to CGRP, but returned to normal levels 1 month earlier than CGRP. Thus, given that the nerve regeneration requires approximately 2 months, galanin may not be a true marker for regenerating axons, but rather a marker for injured axons. These changes may also occur because galanin is involved in the initial stage of axonal re-growth; therefore, its levels decrease in the later stages such as muscle innervation [47].

#### 3.2.3. Pituitary Adenylyl Cyclase-Activating Peptide (PACAP)

PACAP is a neuropeptide member of the secretin family that occurs in two biologically active amidated forms (PACAP38 and PACAP27) close to the vasoactive intestinal peptide (VIP). PACAP was first identified in the ovine hypothalamus and later in the brain and other peripheral organs. Its actions are mediated by two receptors, VPAC1 and VPAC2, which interact with both PACAP and VIP, and by another receptor PAC1, which interacts more specifically with PACAP. PACAP shows increased expression after nerve injury in the sensory ganglia, sympathetic superior cervical ganglion, and rat facial motor nucleus. Following facial nerve injury, PACAP levels also increase greatly, by more than 20-fold, in the facial motor nucleus. Animal experiments showed an increased average axonal length and the number of ranch points in the presence of PACAP [52]. PACAP modulates the expression of neurotrophic factors and their receptors to activate intracellular signaling pathways involved in nerve outgrowth. It also accelerates the reappearance of compound muscle action potentials (CMAPs), improves neuromuscular recovery, stimulates microglial activation, increases the levels of nerve growth factors such as GDNF, and promotes axon myelination. In an animal model to investigate the neurotrophic effect of PACAP, PACAP increased GDNF levels in the facial muscles and PAC1 or VPAC1 was identified at the injured site [54]. Moreover, PACAP provides temporal control of inflammatory immune responses. PACAP receptors are found on microglia and many different types of immune cells. Therefore, immune responses, such as T-cell activation and chemotaxis, are predicted to occur through the action of PACAP. In animal models, have shown that nerve regeneration after facial nerve injury is dependent on T helper cell differentiation by PACAP [55]. In animals lacking PACAP, axon regeneration is delayed and pro-inflammatory cytokine levels increase by 8- to 12-fold. A similar response was also observed in brain stem facial motor neurons. In contrast, PACAP is upregulated by inflammatory mediators derived from T-cells. These results suggest that PACAP is involved in the immune response necessary for nerve regeneration after injury [56].

## 4. Conclusions

More than any other neurological disease, facial paralysis is a disease for which a complete cure is of paramount importance as it greatly reduces the patient’s psychological and emotional quality of life. This need has motivated a variety of studies designed to identify specific mechanisms underlying facial paralysis, discover specific biomarkers, and develop curative treatment methods. It has also been reported that neuropeptides are involved not only in the neurophysiology of the normal facial nerve but also in the regeneration process following facial nerve damage. Among these neuropeptides, alpha-CGRP, galanin, and PACAP are most closely linked to regeneration of injured facial nerves. Although 12 studies have investigated these substances, none were conducted in humans. These neuropeptides, which appear early before changes in the injured cells and their environment, are believed to act as neuromodulators to promote neurotrophic factors involved in nerve regeneration. Moreover, the expression of these neuropeptides depends on the severity of the nerve injury. This reported involvement in regeneration during facial nerve injury suggests the possibility of using neuropeptides as markers of nerve regeneration (Table 3). However, there are some limitations to the study of neuropeptides for facial nerve injury and regeneration. First, only three of the many neuropeptides have been directly studied; thus, research on other neuropeptides in addition to these three is needed. Second, the precise roles and mechanisms of the three proposed neuropeptides have not been elucidated. Third, there is no research on whether neuropeptides influence or are influenced by interactions with other immune cells, inflammatory cells, and/or nerve regeneration substances that appear after facial nerve injury. Fourth, studies on the effects of neuropeptides on the regeneration of facial nerves have not been conducted on human facial nerves. Therefore, future studies should consider the regulation of many neuropeptides and their interactions with other cells in nerve regeneration after injury of facial nerves in humans and in various animal models of facial nerve injury.

## Figures and Tables

**Table 1:** Etiology of facial paralysis.

**Congenital** Mononeural agenesis Congenital facial paralysis Congenital unilateral lower lip palsy Facial paralysis with other deficits Möbius syndrome Hemifacial macrosomia Oculoauriculovertebral dysplasia Poland syndrome Secondary to teratogens Thalidomide Rubella **Polyneuritis** Bell palsy Herpes zoster Guillain-Barré syndrome Autoimmune disease Lyme disease HIV infection Kawasaki disease Tuberculosis **Trauma** Temporal bone fracture Barotrauma Birth trauma Facial laceration Penetrating injury	**Facial Burn** **Radiation injury** **Otitis media** Acute bacterial Chronic bacterial Cholesteatoma **Metabolic and systemic diseases** **Sarcoidosis** **Melkersson-Rosenthal syndrome** **Neurologic disorders** HIV infection Cerebrovascular disorders **Tumors** Schwannoma Glomus tumor Primary parotid tumor Leukemia Histiocytosis Meningioma Rhabdomyosarcoma Metastatic tumor **Recurrent** **Iatrogenic** Mastoid surgery Parotid surgery

**Table 2:** Sources of neuropeptides.

Peptide Source	Neuropeptides
Nervous system, nerve fibers	CGRP, gastrin-releasing peptide, VIP, NPY, Subs P, PACAP, galanin, FRAP, Brain natriuretic peptide
Brain peptides	Leucine enkephalin, methionine enkephalin, Subs P, Gastrin, VIP, Brain GF, brain-derived neurohormone, neurotensin, insulin, glucagon
Cerebral cortex, hippocampus, amygdala	CCK
Hypothalamic peptides	TRH, LHRH, GHIH, CRH, somatostatin, CCK
Pituitary peptides	ACTH, β-endorphin, α-MSH, PRL, LH, TSH, GH, vasopressin, oxytocin, lipotropin
Sympathetic nerves	Noradrenaline, serotonin, NPY
Parasympathetic nerves	Acetylcholine, VIP, NPY, galanin, met-enkephalin
Corneal epithelial cells, endothelial cells, stromal cells, and corneal limbal stem cells, keratocyte	NGF, NT3, NT4, EGF, BDNF, GDNF, Endothelin, NPY, Subs P
Lacrimal gland, macrophages, fibroblasts	EGF
Nasal mucosa glands	PGP, NPY, VIP, Subs P, CGRP, CgA
Nasal mucosal blood vessels	PGP, NPY, VIP, Subs P, CGRP, CgA
Nasopharyngeal mucosa glands	PGP, NPY, VIP, Subs P, CGRP, CgA
Nasopharyngeal mucosa blood vessels	PGP, NPY, VIP, Subs P, CGRP, CgA
Dermal layer of normal skin	Brain natriuretic peptide
Mast cells, keratinocytes, lymphocyte, monocyte, chromaffin cells, and eosinophils	Endothelin, NPY, Subs P
Opioid peptides	Dynorphin, β-endorphin, enkephalins
Circulating peptides	Angiotensin, calcitonin, glucagon

Abbreviations: NPY, neuropeptide Y; Subs P, substance P; CGRP, calcitonin gene-related peptide; VIP, vasoactive intestinal polypeptide; TRH, thyrotropin-releasing hormone; LHRH, luteinizing hormone-releasing hormone; GHIH, growth hormone-inhibiting hormone; CRH, corticotropin-releasing hormone; ACTH, adrenocorticotropic hormone; α-MSH, alpha-melanocyte-stimulating hormone; PRL, prolactin; LH, luteinizing hormone; TSH, thyroid-stimulating hormone; GH, growth hormone; CCK, cholecystokinin; BGF, brain growth factor; PACAP, pituitary adenylate cyclase-activating peptide; FRAP, fluoride-resistant acid phosphatase; NGF, nerve growth factor; NT4, neurotrophin 4/5; EGF, epidermal growth factor; BDNF, brain-derived neurotrophic factor; NT3, neurotrophin-3; GDNF, glial-derived neurotrophic factor; PGP, protein gene product; CgA, chlorogenic acid.

**Table 3:** Studies of neuropeptides expression in facial nerve injury.

Author, Year [Ref.]	Molecule	Experimental Design	Evaluation/Technique	Results	Conclusions
Streit et al., 1989 [51]	CGRP	Rat Facial nerve transection	Structural, molecular changes in facial nucleus Immunohistochemistry, radioimmunoassay	Expression in perikarya dendrites and axons of facial motoneurons: CGRP increase at +15H, maximal levels on Day 6, return to normal after 5–6 weeks. Before glial changes	CGRP: increased facial motoneurons after axotomy
Haas et al., 1990 [57]	CGRP	Rat Facial nerve transection	CGRP expression, localization in facial nuclei Northern blot analysis, in situ hybridization histochemistry (ISHH)	Peak CGRP expression at 16H, return to basal levels at Day 9. CGRP mRNA is expressed in 50% of motoneurons	Early and strong induction of CGRP expression in 50% of motoneurons in the injured facial nucleus
Dumoulin et al., 1991 [49]	CGRP	Rat Facial nerve transected	CGRP expression in regenerating the facial motor nucleus Radioimmunoassay Northern blot analysis	Biphasic five-fold response (Days 3 and 21) in regenerating motoneurons, elevation persists after 45 days No second peak if another resection and ligation of the nerve is performed	CGRP signaling factor: First increase: regulation of astrocyte reaction Second increase: muscle reinnervation
Saika et al., 1992 [45]	Alpha CGRP, beta CGRP, galanin, CCK	Rats Facial nerve crushed or transected	Effect of axonal regeneration on peptide production ÌSHH Comparison control rats, axotomized rats (nerve crush or transection) ISHH	Crushed nerve: Alpha CGRP: single peak and return to normal at 6 weeks Beta CGRP: transient early increase and return to normal at 2 weeks CCK: no response Galanin: delayed response, shorter than after nerve cut Transected nerve: alpha CGRP 2 peaks increase, persist at 8 weeks; beta CGRP: transient early increase than decrease; CCK, galanin: delayed response and persistent elevation	Alpha CGRP, CCK, and galanin increase parallel axon regeneration: trophic effect in motoneuron regeneration Beta CGRP decrease: role in neurotransmission Expression level correlated with nerve recovery. More rapid return to normal after crush compared to the transected nerve
Mohri et al. 2001 [50]	CGRP, c-Jun, GAP-43	Rat Ischemic facial nerve injury	Change of gene expression in facial nuclei after facial nerve ischemia Effect of SOD (superoxide dismutase) on CGRP ISHH	CGRP expression is less elevated and detected earlier after ischemia compared to axotomy. Peaks at Days 3 and 14 (Day 21 in axotomy) SOD, which is a free radical-scavenging enzyme, decreases CGRP expression	CGRP expression changes depending on the extent of nerve damage. Free radicals generated by ischemia partially responsible for ischemic nerve damage and change in gene expression in motoneurons
Burazin et al., 1998 [53]	Galanin GaIRl, GalR2	Rats Facial nerve crushed or transected	temporal changes in galanin, receptors Ga1R1 and Ga1R2 expression in facial motor neurons ISHH	Galanin, GalR2 detected in the facial nucleus on the side of nerve injury but absent on the contralateral side GalR1 not detected GalR2 mRNA increased after 3 days, peaking after 7 days and returning to normal after 14 days (consistent with the time course of axonal regeneration)	Selective upregulation of Ga1R2 after facial nerve injury The receptor may represent an active “autoreceptor” involved in nerve degeneration or regeneration
Makwana, 2010 [46]	Galanin CCRP	Mice Facial nerve transection	Origin, time course, molecular characteristics of sprouting neurites Facial nuclei	Sprouting axons galanin + and CGRP + in and around the facial motor nucleus in white matter, from axotomized motoneuron Delayed appearance of sprouting galanin^+^ (Days 7 to 42)	Galanin and CGRP secreted by facial motor nucleus occur early, with peak expression following injury, later secretion of galanin, coincides with central sprouting and neuronal cell death, neurite-outgrowth enhancing properties of galanin
Kim et al. (2018) [47]	ChAT, CGRP, Galanin, Gephyrin, KCC2	Mice (*n* = 42) Facial nerve transection	Molecule expression facial nucleus, time course of neural functional recovery Immunohistochemistry	Galanin immunolabeling was detected in both axons and cell bodies of FMNs after suturing. Galanin returned to normal level at 1 month (before facial function recovery) Markedly increased CGRP immunolabeling in the genu, nerve root, and FMNs. CGRP expression returned to normal levels when facial functions recovered at Day 60	Changes in CGRP expression during nerve regenerations may be an objective marker of regeneration. However, galanin may be a marker for axon injury
Kimura et al., 2004 [54]	PACAP	Guinea pig Facial nerve transection PACAP injected in injured nerve	Effect of PACAP in GAP-43, GDNF, CMAP. Comparison of PACAP-treated versus non-treated groups	Accelerated reappearance of CMAP Increase and prolonged level of GDNF and myelin	PACAP facilitated the recovery of CMAP, the number of myelinated axons, or both. PACAP can promote nerve regeneration
Armstrong et al., 2004 [55]	PACAP CD4+	Mice immunodeficient (SCID) Facial nerve transection	Effect of CD4 + cells on PACAP induction in motor neurons after facial nerve axotomy Facial nuclei	SCID mice: loss of PACAP gene induction after axotomy CD4 + enriched splenocytes partially restored an upregulation of PACAP	CD4 + lymphocytes play a critical role in the induction of PACAP expression after facial nerve injury. CD4 + cell-dependent induction of PACAP may play a role in nerve/immune cell interaction facilitating nerve regeneration
Suarez et al., 2006 [52]	Galanin, PACAP	Rat Facial nerve transection	Effects of galanin and PACAP on axonal elongation and sprouting	Axonal length and the number of branch points significantly increased in the presence of galanin or PACAP (2–5 μm)	Galanin and PACAP: neurotrophic molecules inducing peripheral axon sprouting. However, it has a limitation due to the stimulation of collateral axon mis-branching
Armstrong et al., 2007 [56]	PACAP	Mice PACAP deficient Facial nerve axotomy and crush	Effects of PACAP on: - facial motor neurons - microglial activation - specific cytokine responses facial nuclei in the brain stem	Deletion of PACAP resulted in: - no differences in motor neuron survival vs. wild-type mice - delayed axon regeneration - reduced numbers of regenerating axons - altered microglial response - amplified inflammatory response in the FMN and nerve site (8- to 12-fold elevation of proinflammatory cytokines TNF-alpha, IL-6, and IFN-gamma)	PACAP: induced nerve regeneration and stimulated microglial activation in the brainstem facial moto nucleus; provided temporal control of the inflammatory immune response

Abbreviations: CGRP, calcitonin gene-related peptide; CCK, cholecystokinin; ISHH, in situ hybridization histochemistry; SOD, superoxide dismutase; GalR1, galanin receptor-1; GalR2, galanin receptor-2; FMN, facial motor nucleus; KCC2, potassium chloride co-transporter 2; PACAP, pituitary adenylate cyclase-activating peptide; GDNF, glial-derived neurotrophic factor; CMAP, compound muscle action potentials; SCID, severe combined immunodeficiency; TNF, tumor necrosis factor; IL, Interleukin; IFN, interferon.
